# Saliva: potential diagnostic value and transmission of 2019-nCoV

**DOI:** 10.1038/s41368-020-0080-z

**Published:** 2020-04-17

**Authors:** Ruoshi Xu, Bomiao Cui, Xiaobo Duan, Ping Zhang, Xuedong Zhou, Quan Yuan

**Affiliations:** 1 State Key Laboratory of Oral Diseases & National Clinical Research Center for Oral Diseases & West China Hospital of Stomatology, Sichuan University, Chengdu, China; 2 State Key Laboratory of Oral Diseases & Human Saliva Laboratory & National Clinical Research Center for Oral Diseases & West China Hospital of Stomatology, Sichuan University, Chengdu, China

**Keywords:** Diagnosis, Public health

## Abstract

2019-nCoV epidemic was firstly reported at late December of 2019 and has caused a global outbreak of COVID-19 now. Saliva, a biofluid largely generated from salivary glands in oral cavity, has been reported 2019-nCoV nucleic acid positive. Besides lungs, salivary glands and tongue are possibly another hosts of 2019-nCoV due to expression of ACE2. Close contact or short-range transmission of infectious saliva droplets is a primary mode for 2019-nCoV to disseminate as claimed by WHO, while long-distance saliva aerosol transmission is highly environment dependent within indoor space with aerosol-generating procedures such as dental practice. So far, no direct evidence has been found that 2019-nCoV is vital in air flow for long time. Therefore, to prevent formation of infectious saliva droplets, to thoroughly disinfect indoor air and to block acquisition of saliva droplets could slow down 2019-nCoV dissemination. This review summarizes diagnostic value of saliva for 2019-nCoV, possibly direct invasion into oral tissues, and close contact transmission of 2019-nCoV by saliva droplets, expecting to contribute to 2019-nCoV epidemic control.

## Introduction

An outbreak of coronavirus disease (COVID-19) is emerging and rapidly spreading worldwide.^1^ A public health emergency of international concern (PHEIC) was declared over COVID-19, which is the sixth time WHO has declared a PHEIC since the International Health Regulations took effect in 2005 (http://www.euro.who.int/en/health-topics/health-emergencies/international-health-regulations/news/news/2020/2/2019-ncov-outbreak-is-an-emergency-of-international-concern).^2^ This new strain of disease was firstly reported in the late December of 2019 and has not been previously identified in human. The novel coronavirus isolated by researchers afterward was named as 2019 novel coronavirus (2019-nCoV).^2^

Coronaviruses are enveloped RNA viruses, and two strains of them—severe acute respiratory syndrome coronavirus (SARS-CoV) and Middle East respiratory syndrome coronavirus (MERS-CoV)—are zoonotic in origin and known to cause fatal respiratory diseases as 2019-nCoV. Due to wide distribution and genomes recombination of coronaviruses, 2019-nCoV is the successive but novel coronavirus and shown to have a higher rate of infection.^3–5^ Early diagnosis of coronavirus and effective prevention of transmission are core tasks in control of 2019-nCoV epidemic.

WHO has claimed that 2019-nCoV spreads primarily through saliva droplets or discharge from the nose (https://www.who.int/health-topics/coronavirus#tab=tab_1). Saliva is secreted 90% from major salivary glands and 10% from minor salivary glands within pH from 6 to 7.^6,7^ Whole saliva is a bio-mixture, which physiologically contains crevicular fluid, desquamated oral epithelial cells, and microorganisms, and may contain blood, respiratory secretions, gastric acid from reflux, and food debris in pathological occasions.^8^ Around 99% of saliva is water and the rest 1% contains a large group of components for the purpose of digesting, taste, buffering, balance of remineralization, and anti-microorganisms.^9^ Oral cavity is an entrance and an outlet of body, and saliva is supposed to play a role in early diagnosis and close contact transmission in infectious diseases. Here, we summarize the reports associated with saliva and 2019-nCoV.

## Diagnostic value of saliva for 2019-nCoV

The officially pathogen detection is the confirmation of 2019-nCoV nucleic acid from throat swabs.^10^ Throat swabs are relatively invasive, induce coughing and cause bleeding occasionally, which may increase risks of healthcare workers infection. Saliva stands at the entry of respiratory system and was also found 2019-nCoV nucleic acid positive.^11–14^ With the nature of noninvasion and less hazard to healthcare workers, saliva specimen collection has the advantages of being more acceptable for patients and more secured for healthcare workers for diagnosis of coronavirus. Till now, three approaches have been reported to collect saliva—coughing out, saliva swabs, and directly from salivary gland duct. In two studies on coughed out saliva, 11 cases out of 12 (91.67%)^11^ and 20 cases out of 23 (86.96%)^12^ COVID-19 patients were 2019-nCoV RNA positive in saliva, respectively. In one study of saliva swabs, half of 15 (50%)^13^ COVID-19 patients were 2019-nCoV RNA positive in saliva. In one study of saliva directly from salivary gland duct, four cases of 31 (12.90%)^14^ COVID-19 patients were 2019-nCoV RNA positive in saliva, three of which were critically ill. Early diagnosis of 2019-nCoV is still difficult, diagnostic value of saliva specimens for 2019-nCoV nucleic acid examination remains limited but promising, which we should still be cautious but expected about.

### Deep throat saliva

A study from To et al. showed that deep throat saliva has high diagnosis rate of 2019-nCoV.^11^ Twelve positive patients were confirmed based on epidemiological history, clinical criteria, and laboratory detection of 2019-nCoV in nasopharyngeal or sputum specimens, and saliva were collected by coughing out a few days after hospitalization.^11^ Using real-time reverse transcription-quantitative polymerase chain reaction by testing the S gene of 2019-nCoV, 11 saliva specimens were positive for 2019-nCoV out of 12 patients (91.67%).^11^ Those 33 patients who are negative for laboratory test of 2019-nCoV were all negative in saliva examination. In addition, six patients offer serial saliva, and five out of them showed a declining trend of virus as hospitalization is going on.^11^ Live virus was detected in three patients of the above six patients by viral culture.^11^

Another study from the same group used self-collected saliva from deep throat by COVID-19 patients, tested 2019-nCoV RNA, and analyzed temporal profile of 2019-nCoV load.^12^ From this study, saliva mixed with nasopharyngeal and bronchopulmonary secretions from deep throat was collected by coughing out in the morning.^12^ Among 23 COVID-19 patients included for this study, 20 cases of their saliva showed detectable 2019-nCoV RNA.^12^ In the temporal profile of viral load, saliva reached the peak of viral load during the first week of symptom onset and then declined.^12^

This group also detected 2019-nCoV RNA of saliva after treatment.^12^ Even if using antibodies against 2019-nCoV, viral RNA could still be detected for 20 days or even longer in deep throat saliva specimens of one third of included patients, suggesting the viral RNA could stay a long period of time instead of dying out after antibody application.^12^ One patient with complete symptom resolved was found 2019-nCoV RNA positive again after 2 days of negative results, suggesting that low levels of 2019-nCoV RNA could still be excreted in saliva even after clinical recovery.^12^ More precisely, whether 2019-nCoV RNA detected in saliva after complete symptom resolved means infectious or shedding virus needs further studies to confirm.

### Saliva in oral cavity

Oral swabs are probably applicable in early detection.^13^ By harvesting oral swabs and testing RNA among 15 COVID-19 patients, Zhang et al. found that half of them (50%) were 2019-nCoV RNA positive in oral swabs, four (26.7%) had positive anal swabs, six (40%) had positive blood test, and three (20%) were serum positive.^13^ Dynamic viral RNA presence in saliva compared with anal swabs were analyzed among 16 patients. Among all swab positive together, most of the positive result was from oral swabs at early stage, while more positive came from anal swabs at late stage of COVID-19, suggesting that oral swabs may indicate early infection of 2019-nCoV but cannot be used as a discharge criteria.^13^

### Salivary gland

To rule out contamination of respiratory secretion, Chen et al. collected saliva directly from the opening of salivary gland and found 2019-nCoV nucleic acid, suggesting that salivary glands were 2019-nCoV infected.^14^ Thirteen cases who were nucleic acid positive by oropharyngeal swab among 31 COVID-19 patients were included, and four of them (12.90%) were positive in saliva.^14^ Three cases of these four were critically ill patents in need of ventilator support, suggesting 2019-nCoV nucleic acid positive in salivary-gland-originated saliva as an indicator of severity of COVID-19.^14^

## Possible direct invasions into oral tissues

In the cycle of infection for most virus, the first step is to attach to the surface and recognize cell surface receptor of the host cell for invasion.^15,16^ With similar external subdomain of receptor-binding domain (RBD), 2019-nCoV spike share same host-cell receptor—angiotensin-converting enzyme II (ACE2)—with SARS-CoV spike, but in a higher affinity than SARS-CoV spike.^17–21^ In another word, cells expressing cell surface receptor ACE2 are susceptible to 2019-nCoV, similar to SARS-CoV. ACE2 was found expressed in lungs, esophagus, ileum, colon, cholangio of liver, and bladder.^22–25^ Consistently, bronchoalveolar-lavage fluid,^2^ nasopharyngeal swabs,^26^ stool,^27,28^ and blood^17^ of COVID-19 patients were RT-PCR-positive for 2019-nCoV. Several studies have shown that salivary gland and tongue express ACE2 receptor, suggesting oral cavity as host for 2019-nCoV to invade.

### Expression of ACE2 in oral tissues

Xu et al. analyzed public bulk RNA-seq from paracarcinoma normal tissues and found expression of ACE2 in oral buccal and gingiva tissue.^29^ This group also analyzed data of single-cell RNA-seq from patients’ oral tissue and found that ACE2 were highly enriched in epithelial cells of tongue, and also in epithelial cells, T cells, B cells, and fibroblasts of oral mucosa.^29^

Saliva is generated in salivary glands and flow through ducts into oral cavity. Liu et al. analyzed rhesus macaques and found ACE2 were also expressed in epithelial cells lining on minor salivary gland ducts,^30^ which could be found in sinonasal cavity, oral cavity, pharynx, larynx, trachea, and lungs, amounting to 800–1000 individuals in total and contributing nearly 1% of saliva a day.^31^ This group also set up animal models by inoculating functional pseudovirus intranasally, and found that ACE2^+^ epithelial cells of minor salivary gland ducts are targeted host cells as early as 48 h after infection.^30^

Besides above evidence from animal study, Chen et al. analyzed data from GTEx, HPA, FANTOM5, and consensus datasets, and revealed the expression of ACE2 receptor in human granular cells in salivary glands.^14^ ACE2^+^ cell in salivary glands could possibly be the target cells of 2019-nCoV and generate infectious saliva in sustained way theoretically.

### Expression of furin on tongue

Furin has been implicated in virus infection by cleaving viral envelope glycoproteins and enhancing infection with host cells.^32^ A furin-like cleavage site in the Spike protein of 2019-nCoV has been identified.^33,34^ Furin is highly expressed in lung tissue, possibly providing a gain-of-function to infectivity of 2019-nCoV.^33,35,36^ Furin expression was detected by immunostaining in human tongue epithelia, and significantly upregulated when squamous cell carcinoma (SCC) occured.^37^ Combined with high expression of ACE2, tongue has high risk of coronavirus infection among oral cavity and SSC even increases the risk once exposed to coronavirus. While it suggests that cells expressing furin have lower restriction for virus entry theoretically, it should still be cautious whether the furin-like cleavage site plays a big role in 2019-nCoV infection.^36^

## Transmission of saliva 2019-nCoV

2019-nCoV transmission occurred within indoor space.^26^ As noted that 2019-nCoV RNA is detected in saliva, whether 2019-nCoV in saliva could be disseminated by long-distance aerosol transmission is concerned by public. WHO has claimed that droplets generated by an infected people by coughing, sneezing, or talking in close contact is the main routine of 2019-nCoV transmission besides touching contaminated surfaces without washing hands (https://www.who.int/news-room/q-a-detail/q-a-coronaviruses#). WHO has updated the definition of close contact—any person within 1 m with a confirmed case at their symptomatic period, starting from 4 days before symptom onset.^38^ However, airborne transmission could also be set up, especially within the same indoor space and aerosol-generating procedure is implemented.

### Size of saliva droplets

Whether droplets can travel long and far along air flow is largely determined by their size.^39^ Most communicable respiratory infections are transmitted via large droplets within short distance or by contacting contaminated surfaces.^40,41^ Large droplets (diameter > 60 μm) tend to quickly settle form the air, so the risk of pathogen transmission is limited to individuals in close proximity to the saliva droplet source.^39^ Small droplets (diameter ≤ 60 μm) may get involved in short-range transmission (distance between individuals less than 1 m). Small droplets are likely to evaporate into droplet nuclei (diameter < 10 μm) in favorable environment, then become potential for long-distance aerosol transmission.^42^

### Generation of saliva droplets by a person

Saliva droplets are generated when breathing, talking, coughing, or sneezing and formed as particles in a mixture of moisture and droplet nuclei of microorganisms.^43^ The amount, distance, and size of saliva droplets varies among people, suggesting the infectious strength and transmission path of saliva droplets differ when same pathogen was contracted.^44^ Three thousand saliva droplet nuclei could be generated by one cough, which nearly equals to the amount produced during a 5-min talk.^43^ Around 40,000 saliva droplets reaching several meters in air can be generated by one sneeze.^43,45^ One normal exhalation can generate saliva droplets reaching the distance of 1 m in air.^43^ Large saliva droplets with more mass tends to fall ballistically to the ground and small saliva droplets travel like a cloud over longer distance by air flow.^39,43,45^

### Environment-dependent saliva aerosols transmission

Aerosols are suspension of particles in air, liquid, or solid, within size from 0.001 to above 100 μm.^39^ Infectious aerosols contain pathogens.^39^ Long-distance aerosol transmission is determined by sufficiently small infectious droplets, being almost indefinitely airborne and transmitted at a long distance (distance between individuals more than 1 m).^39^ Aerosol transmission is well accepted in infection of tuberculosis, measles, and chickenpox, and other infectious agents may behave as airborne transmission in a favorable environment or opportunistically, such as SARS-CoV, influenza virus, and adenovirus.^40,46^ Opportunistically airborne transmission is a mode that infectious agents not only have transmission routines by contacting and droplets but also can reach distant susceptible hosts under restricted conditions by fine-particle aerosols in favorable environments.^40^ It is possible when aerosol-generating procedure is implemented, such as dental practice, that 2019-nCoV could possibly spread in airborne transmission.^38,47^

Whether saliva droplets can become truly long-distance aerosol transmission is determined by how long the saliva droplets can reside in the air (physical decay), how long the pathogen in saliva droplets remain infectious (biological decay), and whether theses infectious saliva droplets can be acquired by another person (acquisition).^40^ In terms of physical decay, saliva droplets evaporate fast into reduced mass in dry air, tending to stay longer along with air flow.^39^ The composition of droplet nuclei determines its terminal size.^39^ For droplets with slow biological decay, temperature differences and opened door set up droplets exchange along with air flow.^48^ Biological decay is determined by dehydration, exposure to ultraviolet and chemicals.^39^ Only hardy organism such as *M. tuberculosis* can survive long in air to form long-distance transmission.^49^ A recent review summarized that coronavirus stay vital on surfaces of metal, glass, or plastic for up to 9 days, but no solid evidence has been found how long in air.^50^ The coronavirus on inanimate surfaces could be efficiently inactivated by 0.1% sodium hypochlorite, 62–71% ethanol, or 0.5% hydrogen peroxide within 1 min as summarized in the literature.^50^

So far, no solid evidence to consistently support that 2019-nCoV in saliva droplets can keep vital along air flow for very long time. Liu et al. collected 35 aerosol samples from three areas in two hospitals of Wuhan, and tested 2019-nCoV RNA by droplet digital polymerase chain reaction.^51^ They found that patient area had low or even undetectable aerosol 2019-nCoV RNA, but deposition aerosol were tested positive, suggesting that not much vital virus in air flow but tend to deposit to the floor, which is similar to movements of large saliva droplets as noted previously.^51^ In medical staff area, airborne 2019-nCoV RNA concentration was decreased after patients reduced and sanitization rigorously implemented.^51^ In public area, accumulation of crowds increased airborne 2019-nCoV RNA concentration from undateable level.^51^

### Acquisition of infectious saliva aerosols

For acquisition of infectious saliva droplets by a susceptible host, infectious saliva droplets could land in month, eyes, or be inhaled into lungs directly.^26,52^ A case report shows that 2019-nCoV infection occurred in a fever clinic when a susceptible person wore an N95 mask covering mouth and nose without eyes protected, suggesting a transmission to eyes.^53^ It is also reported that SARS-CoV is predominantly transmitted by contacting eye, mouth, or nose.^54^ Respiratory virus could lead to respiratory infections of another person through inducing ocular complications.^55^ Exposed mucous membranes increased risk of virus transmission by a SARS-CoV study, and close exposure to an infected person increases the chance of infection.^55^ A previous study confirmed that infection of SARS-CoV was reduced to a certain degree by wearing surgical masks of susceptible healthcare workers.^56^

## Comparison of saliva 2019-nCoV and SARS-CoV

2019-nCoV, which is also named as SARS-CoV-2,^57^ shares about 79% nucleotide sequence similarity with SARS-CoV.^5,17,58–61^ SARS-CoV have a higher mortality rate, while 2019-nCoV spreads much faster.^12^ The similarities and differences of saliva are summarized as follows in terms of diagnosis value of saliva, direct invasion to oral tissues, and saliva droplet transmission between SARS-CoV and 2019-nCoV, hopefully explaining the faster transmission speed of 2019-nCoV (Table 1).

**Table 1:** Comparison of 2019-nCoV and SARS-CoV in terms of saliva

Items	2019-nCoV	SARS-CoV
Diagnostic value of saliva	(1) Early detection of viral RNA in saliva. (2) Viral peaks at onset of symptoms. (3) Salivary gland originated virus RNA is associated with severe COVID-19.	(1) Early detection of viral RNA in saliva. (2) Viral peaks 10 days after symptoms. (3) A high initial SARS-CoV load was associated with death.
Direct invasion to oral cavity	(1) ACE2 receptor on host cells of tongue and salivary gland. (2) A furin-like cleavage site is peculiar in the S protein of 2019-nCoV.	ACE2 receptor on host cells of tongue and salivary gland.
Infectious saliva droplets	Possible opportunistically airborne transmission.	Opportunistically airborne transmission.

For the diagnostic value of saliva in coronavirus infection, high expression level of SARS-CoV RNA was detected in saliva specimens from 17 SARS patients, four of which had not yet lung lesion, suggesting value of early diagnosis of saliva, similarly to 2019-nCoV.^62^ A previous animal study on early events of SARS-CoV infection showed that SARS-CoV was detected in oral swabs before blood test turned positive on second day after viral challenge through nasal cavity.^30,63^ The viral load profile in saliva of 2019-nCoV nearly peaks at the time of symptom onset, while SARS-CoV peaks at around 10 days after symptoms.^64–67^ The high viral load of 2019-nCoV suggests it can be transmitted even if symptom is mild or less obvious. Older age was associated with higher SARS-CoV in saliva, and a high initial SARS-CoV load was associated with death.^68,69^ 2019-nCoV RNA could be detected in saliva for 20 days or even longer, and the prolonged detection of viral RNA also exist in SARS-CoV and MERS-CoV infection.^64–67^

Despite 2019-nCoV and SARS-CoV share ACE2 receptor on host cells, which are found in salivary gland and tongue tissues,^23–25,29,53^ 2019-nCoV is likely more infectious than SARS-CoV possibly due to lower RBD-ACE2 binding free energy and more flexible RBD of 2019-nCoV than that of SARS-CoV.^70^ Compared with SARS-CoV, a furin-like cleavage site is peculiar in the S protein of 2019-nCoV, which could theoretically be cleaved by furin expressed in tongue tissues.^33,34^

For saliva droplets as transmission routine in coronavirus infection, a retrospective cohort study of SARS-CoV transmission reported that students who were in the same cubicle with the SARS patient contracted SARS-CoV, telling us that proximity to SARS patients increases chances of SARS-CoV infection.^52^ Virus-laden droplets were found in a study as a routine of transmission in SARS epidemics.^71,72^ van Doremalen et al. evaluated the stability of 2019-nCoV and SARS-CoV in aerosils using a Bayesian regression model, and found that 2019-nCoV remained viable ins aerosols throughout 3-h experiment duration, which is similar to SARS-CoV.^73^ SARS-CoV dissemination belongs to opportunistically airborne transmission.^40,74^ 2019-nCoV also possibly belong to the same transmission type, dissemination of virus occurs if individuals are exposed to a high concentration of infectious aerosols in comparatively sealed space for long time.^75^

## Prospective

It seems that the diagnostic value of saliva depends on how saliva specimens are collected. Saliva from deep throat (91.67 and 86.96% corresponding to two studies), from oral cavity (50%), or from salivary glands (12.90%) indicates a diagnostic tendency of decreased positive rate of 2019-nCoV RNA among COVID-19 patients.^11–14^ For clinical application in need of high positive rate of virus detection, saliva from deep throat has the highest positive rate, which may stand for early diagnosis of COVID-19. Saliva directly from saliva glands ducts is associated with severe COVID-19 and possibly could be a predictive and noninvasive test for severed patients. Whether 2019-nCoV RNA in saliva equals to infectious saliva or a condition of shedding vital virus is still lacking evidence. Even if diagnosis by saliva is noninvasive and less hazardous compared with throat swabs, comprehensive diagnosis should be supported by complete information of symptoms, epidemiological history, and analysis of multiple clinical examinations.

Besides lungs, oral tissue is possible to be directly invaded theoretically due to expression of ACE2 receptor and furin enzyme.^14,29,30,37^ About half of the victims reported symptoms of dry mouth and amblygeustia.^14^ These symptoms probably came from dysfunction of tongue expressing ACE2 and furin, and salivary gland expression ACE2. However, there is no histopathological evidence to support the direct invasion of 2019-nCoV to oral tissue so far. While it suggests that cells expressing ACE2 and furin have lower restriction for virus entry theoretically, the molecular mechanism of 2019-nCoV infection is not yet fully unfolded and we should still be cautious about and not overstating the current virus-invade-host theory.

**Fig. 1:**
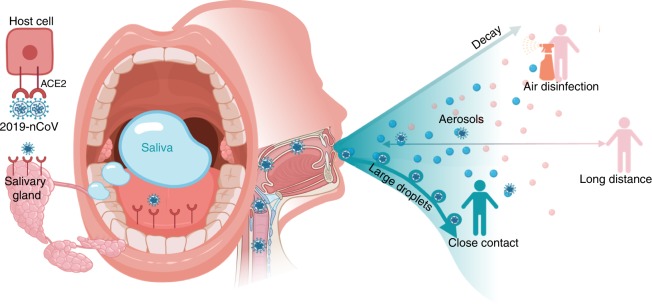
Potential diagnostic value of saliva and transmission of 2019-nCoV. Possibly combing to host-cell receptor of ACE2 expressed in salivary glands and tongue, 2019-nCoV is detected in saliva. Combined with infectious fluids from respiratory system, 2019-nCoV via large saliva droplets sets up short-distance transmission and hardly form long-distance aerosol transmission outdoors due to complicated physical and biological decay. Prevention of droplets formation, implementation of air disinfection, and blockage of droplets acquisition could possibly slow down 2019-nCoV dissemination

Saliva is a common and transient medium for virus transmission. Among saliva droplets with different sizes generated by breathing, talking, and sneezing, large droplets easily fall onto the floor and only set up short-distance transmission.^39^ Saliva could form aerosols and reach a distant host along air flow when in a favorable environment.^39^ So far, no solid evidence supports that SARS-nCoV or 2019-nCoV can survive in air outdoors for long time to set up long-distance aerosol transmission. Therefore, wearing masks to prevent formation of infectious saliva droplets projecting to the air, thorough disinfection of indoor air to block dissemination of infectious saliva droplets, and keep a distance with people not to acquire infectious saliva droplets could slow down 2019-nCoV epidemic to a certain degree (Fig. 1).
